# Plasmablastic lymphoma mimicking primary ovarian, tubal or peritoneal cancer: a case report and literature review

**DOI:** 10.1007/s12672-025-02186-y

**Published:** 2025-04-08

**Authors:** I-Ning Cheng, Chen Chang, Hong-Ming Tsai, Ya-Ting Hsu, Yu-Fang Huang

**Affiliations:** 1 Department of Obstetrics and Gynecology, National Cheng Kung University Hospital, College of Medicine, National Cheng Kung University, 138, Sheng-Li Road, Tainan, 704302 Taiwan; 2 Department of Obstetrics and Gynecology, Cathay General Hospital, Taipei, 10630 Taiwan; 3 Department of Pathology, National Cheng Kung University Hospital, College of Medicine, National Cheng Kung University, Tainan, 704302 Taiwan; 4 Department of Medical Imaging, National Cheng Kung University Hospital, College of Medicine, National Cheng Kung University, Tainan, 704302 Taiwan; 5 Division of Hematology, Department of Internal Medicine, National Cheng Kung University Hospital, College of Medicine, National Cheng Kung University, 138, Sheng-Li Road, Tainan, 704302 Taiwan

**Keywords:** Plasmablastic lymphoma, Ovarian cancer, Primary ovarian lymphoma, Non-Hodgkin lymphoma, Lymphoma

## Abstract

**Introduction:**

Plasmablastic lymphoma (PBL), an extremely aggressive B-cell non-Hodgkin lymphoma, is known to be associated with immunosuppression. PBL with primary peritoneal or ovarian involvement is extremely rare. Here, we report a case of PBL in an immunocompetent woman, which involved the right ovary, peritoneum, cervical lymph nodes, and bone marrow.

**Case presentation:**

A 42 year-old immunocompetent premenopausal woman presented with abdominal dullness and distension. Imaging studies revealed the presence of ascites, ovarian mass, peritoneal seedings, and generalized lymphadenopathy. Laparoscopic biopsies of the right adnexal tumor, omental cake, and peritoneal seedings were performed. Poorly differentiated carcinoma was suggested from initial intraoperative frozen sections. The conclusive histological and immunohistochemical diagnosis was however that of PBL. Bone marrow involvement was also confirmed. The patient was treated with dose-adjusted etoposide, prednisone, vincristine, cyclophosphamide, and doxorubicin plus daratumumab and bortezomib. Haploidentical peripheral blood stem cell transplantation (halpo-PBSCT) was performed to achieve better disease control. The patient showed sustained partial response for six months.

**Conclusion:**

The diagnosis of immunocompetent PBL with intraperitoneal spread before surgery and during intraoperative cryosections remains challenging. Intraperitoneal biopsy and delayed cytoreduction can prevent unnecessary tumor resection. However, a definitive treatment modality is yet to be established. Our patient responded to chemotherapy plus targeted therapy, followed by halpo-PBSCT, leading to a 6-month partial response. Our experience provides additional information on pretreatment evaluation and therapeutic considerations for patients with PBL.

## Introduction

Plasmablastic lymphoma (PBL), an overly aggressive B-cell non-Hodgkin lymphoma, is frequently associated with immunosuppression, particularly following human immunodeficiency virus (HIV) infection [1]. PBL typically presents as a mass in one or more extranodal sites, usually in the oral cavity or gastrointestinal tract. PBL with primary involvement of the ovaries, cervical lymph nodes, and bone marrow is unusual in immunocompetent women. HIV infection seems to result in the loss of immune control over oncogenic viral infections, such as Epstein–Barr virus (EBV) infection, which is detected by the positive expression of Epstein–Barr encoding region (EBER). Moreover, EBV infection is strongly associated with HIV infection in 80% of immunosuppressive cases. To the best of our knowledge, this is the first case of an aggressive, advanced disease mimicking late-stage epithelial ovarian cancer (EOC), tubal cancer (TC), or primary peritoneal cancer (PPC).

## Case presentation

A 42 year-old immunocompetent woman, G0P0, had type II diabetes mellitus before presenting to our gynecologic department with a 3-week history of abdominal dullness and distension. There was no associated fever, chest pain, nausea, vomiting, diarrhea, dysuria, poor appetite, or decreased body weight. Abdominal ultrasonography revealed the presence of bilateral adnexal masses and moderate ascites. Upon abdominal computed tomography (CT), ovarian carcinoma with peritoneal carcinomatosis and pelvic and para-aortic lymphadenopathies were suspected. Elevated cancer antigen (CA)-125 levels (249 U/mL) were also detected. Levels of CA-199 and carcinoembryonic antigen were within normal limits.

**Fig. 1:**
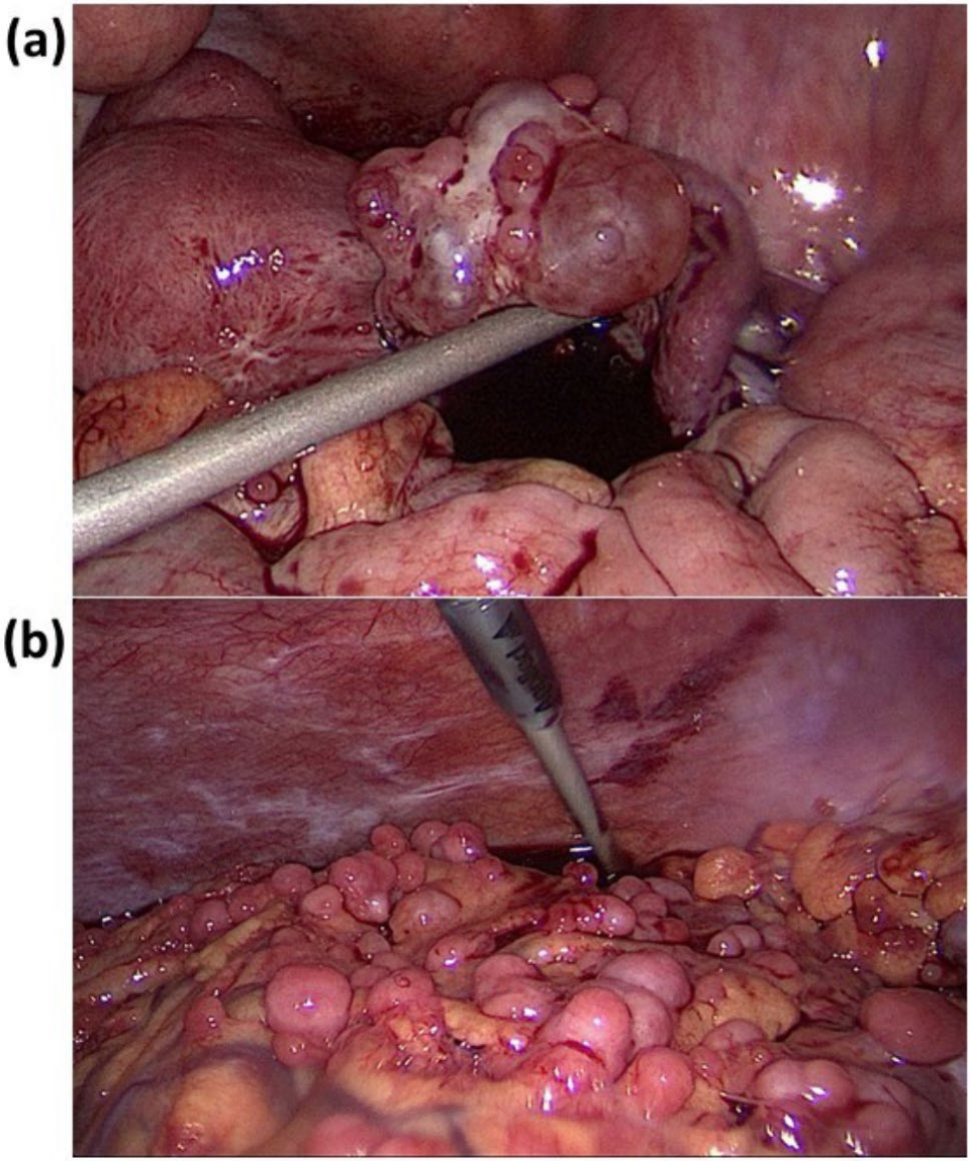
Intraoperative findings. **a** Bilateral ovarian tumors with multiple tumor seedings over the pelvic peritoneal cavity; **b** disseminated tumors over the omentum, subdiaphragm, and upper abdominal peritoneal cavity

**Fig. 2:**
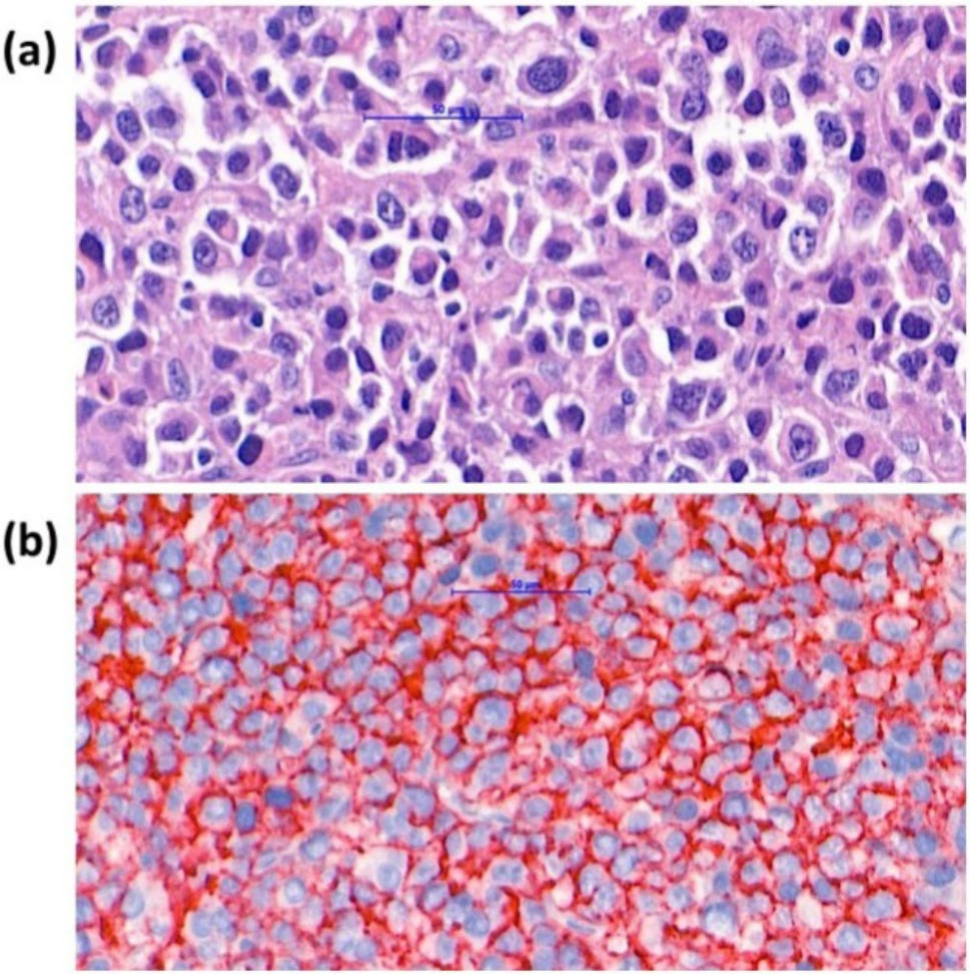
Microscopic characteristics of plasmablastic lymphoma of the ovary. **a** Plasmacytoid tumor cells with eccentric nuclei, prominent nucleoli and perinuclear halo (H&E stain, 400X); **b** CD 138-positive tumor cells (Immunohistochemistry, 400X)

Diagnostic laparoscopy revealed the presence of right ovarian tumors with multiple disseminated tumors in the peritoneal cavity, subdiaphragm, falciform ligament, omentum, and abdominal wall (Fig. 1a and b). The right ovary, right tube, peritoneal seeding tumors, and a segment of the omental cake were excised. Intraoperative cryosections suggested a poorly differentiated carcinoma. EOC, TC, and PPC were also suspected. Substantive histological assessment on routine hematoxylin and eosin sections showed aggregates and sheets of plasmacytoid tumor cells with eccentric nuclei, prominent nucleoli and perinuclear halo in the right ovarian and peritoneal tumors (Fig. 2a). Immunohistochemically, the tumor cells were positive for cluster of differentiation (CD)138, CD38, kappa, and c-MYC antibodies but negative for lambda, cytokeratin, CD3, CD20, CD56, human herpesvirus-8, and anaplastic lymphoma kinase antibodies (Fig. 2b). The EBER expression in situ hybridization was negative. The Ki-67 proliferation index was high (80–90%). Combining the histomorphological findings and immunophenotype, a diagnosis of PBL involving the right ovary, peritoneum, and abdominal wall was established.

**Fig. 3:**
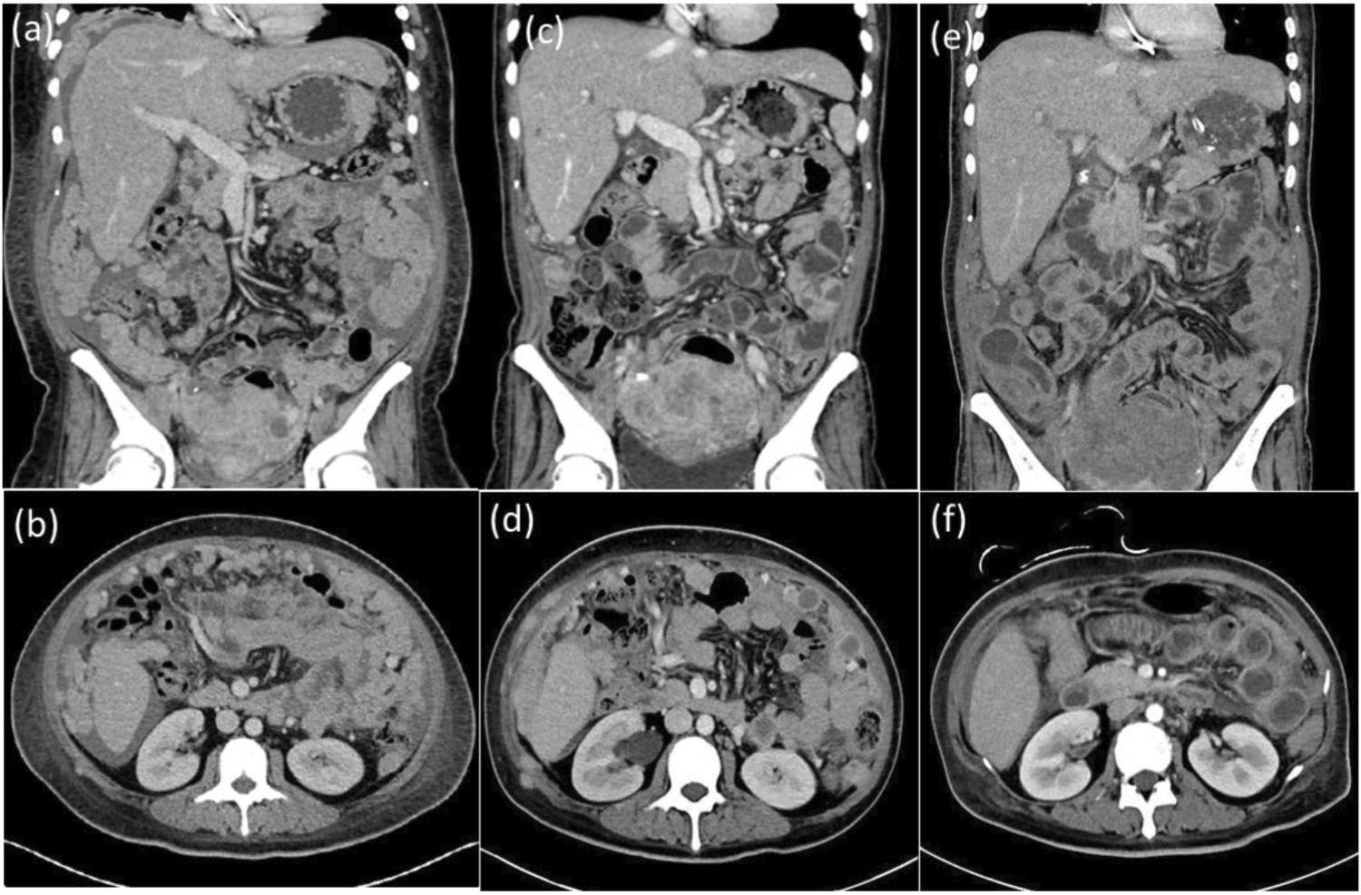
Coronal view **a** and **c** and transverse view **b** and **d** of abdominal and pelvic computed tomography. **a** and **b** Before treatment: bilateral adnexal masses with diffuse lymphadenopathies in the mesentery, multiple peritoneal tumors over the omentum, and peritoneum. **c** and **d** After treatment: tumor shrinkage and partial regression of plasmablastic lymphoma after chemotherapy plus target therapy; **e** and **f** showed stationary lymphadenopathies with interval regression after halpo-PBSCT

The patient was immediately referred to a hematologist for further evaluation and treatment. HIV test results were negative. Bone marrow aspiration and biopsy confirmed the bone marrow involvement. Moreover, whole-body CT revealed lymphadenopathy in the right supraclavicular, bilateral internal mammary, mediastinal, cardiophrenic, pericardial, and para-aortic regions (Fig. 3a and b). The patient was classified as Ann Arbor stage IV and, hence, underwent five cycles of dose-adjusted (DA) chemotherapy combinations (etoposide, vincristine, doxorubicin, cyclophosphamide, and prednisone [EPOCH]) plus an anti-CD38 antibody (daratumumab) and a proteasome inhibitor (bortezomib). A partial response was achieved (Fig. 3c and d), with regression of the abdominal distension and lymphadenopathies observed after two cycles of treatment. However, interval progression was noted in the bilateral abdominal walls, whereas peritoneal lesions and metastatic lymphadenopathies remained stationary after four cycles of treatment. The response status was based on the response evaluation criteria in lymphoma (RECIL) classification [18], revealing that the sum of the longest diameter of target lesions was reduced by >30%; however, the lesion did not disappear completely. The patient received haploidentical peripheral blood stem cell transplantation (haplo-PBSCT) using stem cells procured from her sibling brother with their consents. This procedure aimed to enhance disease control and leverage the graft-versus-lymphoma effect. A myeloablative conditioning regimen, consisting of fludarabine and busulfan, was administered prior to the transplantation. The procurement of stem cells adhered to ethical guidelines. The patient achieved a sustained partial response for six months, with regression of intraperitoneal diseases and lymphadenopathies observed on CT (Fig. 3e and f).

## Discussion

PBL with ovarian involvement is rare, with only five case reports documented in the literature (Table 1) [2–6]. As seen in our patient, four of five patients did not have an HIV infection and their clinical manifestations varied according to the symptoms and extra-ovarian sites involved. However, the efficacy of anticancer therapy and treatment outcomes in these patients remain inconclusive.

**Table 1:** Plasmablastic lymphoma patients with ovarian involvement in literature

Author name [Ref No.]	Clinico-pathological characters	Involved sites	Pathology, IHC	Treatment and outcome
Shruthi et al., [2]	Age of 25; fever, cough, and breathlessness for two weeks	Bilateral adnexa, bone marrow, enlarged para-aortic and mesenteric lymph nodes	CD45 focally positive, CD79a positive, CD38 diffusely positive, MUM1 positive; CK, CD5, CD20 negative; ALK, CD30, PAX8, and inhibin negative	One cycle of CHOP-R chemotherapy. The patient died due to SARS CoV-2 pneumonia
Kale et al., [3]	Age of 46; HIV (+); oral ulcers, dysphagia, and epigastric pain	Right adnexa; lung nodule (lung adenocarcinoma)	Not applicable	Six cycles of CHOP chemotherapy followed by involved site radiotherapy. No treatment outcome was recorded
Hadžisejdić et al., [4]	Age of 19; HIV (–); generalized lymphadenopathy	Right ovarian mature cystic teratoma with PBL involvement	CD79a, PAX5, MUM1, CD38, CD138 positive; CD20, CD10, Bcl-6, ALK, cyclin D1, CD56 negative	Four cycles of dose-adjusted (DA)-EPOCH as well as intrathecal EPOCH. Autologous peripheral blood stem cell transplantation, followed by 4 cycles of alternating modified CODOX-M and IVAC. The patient died of disease progression and multiple organ failure
Suvarna et al., 2017 [5]	Age of 37; HIV (–); abdominal distension for 1 year	Bilateral adnexa	CD56, BCL2, CD43 and CD79 positive; CD20, PAX5, CD3, CD4, Bcl-6, P53, LCA/CD45 and HHV-8 negative	Eight cycles of CHOP chemotherapy. The patient remained remission after 18 months of follow-up
Avilés-Salas, et al., 2022. [6]	Age of 27; HIV (+); abdominal pain and tingling sensation, with paresthesia and dysesthesias in the left pelvic limb	Ovary, cervix and soft thigh tissues involvement	Not applicable. (Article in Spanish)	Not applicable
Our case	Age of 42; HIV (–); 3 week abdominal dullness and distension	Bilateral adnexa, peritoneal tumors, omentum, pelvic and paraaortic lymph nodes	CD138, CD38, kappa, and cMYC positive; lambda, CK, CD3, CD20, CD56, HHV-8, and ALK, negative	Five cycles of DA-EPOCH plus daratumumab and bortezomib. Haploidentical PBSCT was conducted. The patient showed sustained partial response for 6 months but encountered GVHD after the transplantation

ALK: anaplastic lymphoma kinase; Bcl: B-cell lymphoma; CD: cluster of differentiation; CK: cytokeratin; HHV-8: human herpesvirus-8; HIV: human immunodeficiency virus; IHC: immunohistochemistry; MUM: multiple myeloma oncogene; PAX: Paired-box; PBL: plasmablastic lymphoma; DA-EPOCH: dose-adjusted etoposide, prednisone, vincristine, cyclophosphamide, doxorubicin; CODOX-M/IVAC chemotherapy: cyclophosphamide, vincristine, doxorubicin, high-dose methotrexate/ ifosfamide, etoposide, and high-dose cytarabine; CHOP chemotherapy: cyclophosphamide, doxorubicin, vincristine, prednisolone; PBSCT: peripheral blood stem cell transplantation; GVHD: graft-versus-host disease

Preoperative assessments, including CT imaging, cannot differentiate PBL from carcinoma in patients with clinical characteristics similar to those of primary ovarian, tubal, or peritoneal cancer. Typically, PBL is diagnosed by a hematopathologist with appropriate immunohistochemical studies using permanent sections, although diagnosis using a frozen section procedure can be challenging. In the current case, small aggregates of tumor cells were dispersed on a fibrous background with blurred, irregular cell borders owing to freezing artifacts. These features imparted a false impression of a cell arrangement with cohesiveness and an epithelioid shape, mimicking carcinoma. A common differential diagnosis for aggressive large-cell lymphoma is poorly differentiated carcinoma. Laparoscopic intraperitoneal biopsy may be indicated to avoid unnecessary cytoreduction in cases of PBL mimicking EOC/TC/PPC. In general, the cytomorphology of the bone marrow and peripheral blood could be valuable for hematolymphoid neoplasm diagnosis using frozen sections and during consultation [11], but this typically requires a high index of suspicion. Nevertheless, a permanent pathological section is a reliable and necessary test for a definite diagnosis and subtyping by a hematopathologist. Once PBL is diagnosed, the patient should be referred to a hematologist as soon as possible for comprehensive evaluation and further treatment.

Currently, there are no definitive treatment regimens or guidelines based on sufficient research owing to the rarity of this condition. The National Comprehensive Cancer Network guidelines on B-cell lymphomas recommend more intense regimens for PBL and suggest DA-EPOCH as the preferred alternative to EPOCH [1, 12]. In patients with aggressive-stage PBL, daratumumab, a monoclonal CD38 antibody, combined with chemotherapy, reportedly achieved favorable response rates (54–58%) [7, 8]. Additional proteasome inhibitors (bortezomib) also achieved promising results (71.4–100%) [9, 10].

Recent studies have suggested the use of autologous hematopoietic stem cell transplantation in patients with PBL [14, 15]. Allogeneic hematopoietic stem cell transplantation (allo-HSCT) may be an alternative for relapsed and refractory disease. Broccoli et al. reported a case of nodal HIV-unrelated PBL that achieved complete remission for a minimum of 10 years after intensive therapy, which included double autologous stem cell transplantation [16]. Rong et al. reported an HIV-negative relapsed patient who achieved remission after allo-HSCT [17]. Among patients who are candidates for allo-HSCT, haplo-HSCT is currently considered an alternative transplantation protocol [13], although a stronger graft-versus-tumor effect may be experienced. Given the aggressiveness and poor prognosis of PBL in our case, the treatment strategy comprised intensive chemotherapy (DA-EPOCH) with concurrent targeted therapies (daratumumab and bortezomib). This approach was supported by growing evidence of its efficacy. To address residual disease and enhance disease control, the patient underwent haplo-PBSCT following a myeloablative conditioning regimen. While the patient achieved a sustained partial response six months post-transplantation, further follow-up is required to evaluate long-term outcomes.

Moreover, high expression of programmed cell death 1 (PD-1)/PD1 ligand (PD-L1) was detected in PBL, and the PD-1/PD-L1 pathway was abnormally activated. Partial response in refractory PBL has been reported after PD-1 blockade; nevertheless, the efficacy of PD-1 inhibitors remains unclear [14, 19]. The combination protocol that leads to the most promising effects on prolonged survival should be clarified in the future.

## Conclusion

Although PBL is extremely rare in female patients with suspected peritoneal carcinomatosis, it should be considered a differential diagnosis, given its extreme aggressiveness and poor prognosis. PBL may mimic EOC/TC/PPC on frozen sections; permanent pathological sections with specific immunohistochemical examinations following laparoscopic biopsy can prevent excessive surgical resection. This case report emphasizes the importance of a multidisciplinary approach in managing PBL, particularly in those with atypical presentations. It also highlights the challenges in early diagnosis and the unmet need to develop the optimal treatment protocol with combined chemotherapy, targeted therapies, and stem cell transplantation concurrently or sequentially. Future investigations seeking the most beneficial combination regimens with less adverse events should be conducted to improve disease control in patients with PBL.

## Data Availability

Data sharing is not applicable to this article as no datasets were generated or analyzed during the current study.

## Abbreviations

BMT: Bone marrow transplantation
CD: Cluster of differentiation
DA-EPOCH: Dose-adjusted etoposide, prednisone, vincristine, cyclophosphamide, doxorubicin
HIV: Human immunodeficiency virus
PBL: Plasmablastic lymphoma
halpo-PBSCT: Haploidentical peripheral blood stem cell transplantation
ASCT: Autologous hematopoietic stem cell transplantation
allo-HSCT: Allogeneic hematopoietic stem cell transplantation
GVHD: Graft-versus-host-disease
PD-1: Programmed cell death 1
PD-L1: Programmed cell death ligand 1
EOC: Epithelial ovarian cancer
TC: Tubal cancer
PPC: Primary peritoneal cancer
